# Supplementary material for: Mechanisms governing the pioneering and redistribution capabilities of the non-classical pioneer PU.1
Source: Nat Commun. 2020 Jan 21;11:402. doi: 10.1038/s41467-019-13960-2 (PMC6972792; doi:10.1038/s41467-019-13960-2)
Supplement: Supplementary file 7 — Source data [file 41467_2019_13960_MOESM7_ESM.zip › Source_Data/FigureS2/FigureS2B_MotifScanOutput/KmeansCluster6/homerResults/motif23.similar.html]

motif23

## Information for motif23

A
G
T
C
A
C
G
T
A
T
G
C
A
C
G
T
A
G
T
C
A
G
T
C
A
G
T
C
A
C
G
T
A
G
T
C
A
G
T
C
C
G
A
T
C
A
T
G
G
A
T
C
  
Reverse Opposite:  

C
T
A
G
G
T
A
C
C
G
T
A
C
T
A
G
T
C
A
G
C
G
T
A
A
C
T
G
C
T
A
G
C
T
A
G
C
G
T
A
A
T
C
G
C
G
T
A
A
C
T
G
  

|  |  |
| --- | --- |
| p-value: | 1e-43 |
| log p-value: | -1.004e+02 |
| Information Content per bp: | 1.776 |
| Number of Target Sequences with motif | 144.0 |
| Percentage of Target Sequences with motif | 4.37% |
| Number of Background Sequences with motif | 401.5 |
| Percentage of Background Sequences with motif | 0.93% |
| Average Position of motif in Targets | 105.0 +/- 43.7bp |
| Average Position of motif in Background | 92.3 +/- 88.3bp |
| Strand Bias (log2 ratio + to - strand density) | 0.6 |
| Multiplicity (# of sites on avg that occur together) | 1.00 |
| Motif File: | file (matrix) reverse opposite |

### Similar de novo motifs found

|  |  |  |  |  |  |  |  |
| --- | --- | --- | --- | --- | --- | --- | --- |
| Rank | Match Score | Redundant Motif | P-value | log P-value | % of Targets | % of Background | Motif file |
| 1 | 0.842 | A T G C A C G T A G T C A G C T A T G C A G C T A G T C C G A T A G T C A G T C A C G T T A C G A G T C A G C T | 1e-34 | -79.776270 | 3.19% | 0.62% | motif file (matrix) |
| 2 | 0.824 | A G C T A G T C G A C T A G C T G A T C G A T C G C A T A T G C A G T C G C A T C A T G A T G C G A C T | 1e-30 | -70.116548 | 3.40% | 0.81% | motif file (matrix) |
| 3 | 0.811 | C A G T G T A C C G A T A G T C G A C T A T G C G A C T G A T C A G T C A C G T T C A G G T A C | 1e-26 | -61.988189 | 2.49% | 0.49% | motif file (matrix) |
